# Strain and Vibration in Mesenchymal Stem Cells

**DOI:** 10.1155/2018/8686794

**Published:** 2018-01-09

**Authors:** Brooke McClarren, Ronke Olabisi

**Affiliations:** Department of Biomedical Engineering, Rutgers University, 599 Taylor Rd, Piscataway, NJ 08854, USA

## Abstract

Mesenchymal stem cells (MSCs) are multipotent cells capable of differentiating into any mesenchymal tissue, including bone, cartilage, muscle, and fat. MSC differentiation can be influenced by a variety of stimuli, including environmental and mechanical stimulation, scaffold physical properties, or applied loads. Numerous studies have evaluated the effects of vibration or cyclic tensile strain on MSCs towards developing a mechanically based method of differentiation, but there is no consensus between studies and each investigation uses different culture conditions, which also influence MSC fate. Here we present an overview of the response of MSCs to vibration and cyclic tension, focusing on the effect of various culture conditions and strain or vibration parameters. Our review reveals that scaffold type (e.g., natural versus synthetic; 2D versus 3D) can influence cell response to vibration and strain to the same degree as loading parameters. Hence, in the efforts to use mechanical loading as a reliable method to differentiate cells, scaffold selection is as important as method of loading.

## 1. Introduction

In tissue engineering and regenerative medicine, mesenchymal stem cells (MSCs) are often preferable to fully differentiated cells, which are limited in supply and do not multiply as rapidly or to as great an extent [1]. MSCs can proliferate for numerous passages. The MSC response to tensile strain and vibration has been researched using various scaffolds and stimulation parameters. Typical MSC responses to various mechanical loading include differentiation into osteocytes and chondrocytes, often guided by the presence of growth factors and calcium. Cell responses have also been guided by the microenvironment, whether cells are in their native environment, a transplanted* in vivo* environment, or cultured using tissue culture plastic, 2D scaffolds, or 3D scaffolds. Even the choice of scaffold material has an impact, that is, whether the scaffold is derived from natural or synthetic material. Although it is well known that MSCs respond to mechanical loading, it is not known how to best load these cells to achieve the desired differentiation. Identifying which combination of scaffold and loading protocol are associated with which MSC fate may permit researchers to reliably control differentiation without using differentiation media. Bending, tension, mechanical compression, hydrostatic compression, fluid shear, and vibration are all experienced by MSCs* in vivo*, as such their effects on MSCs have been explored extensively* in vitro.* When examining the aforementioned loading conditions, tensile strain of tissues is perhaps the easiest to measure* in vivo*, while vibration is the easiest to apply* in vivo*. Thus, for tensile strain and vibration, it is possible to compare their effects when applied* in vivo* versus* in vitro*. Therefore, this review focuses on the effect of tensile strain and vibration on the fate of MSCs in a variety of culture environments.

## 2. Common Methods to Differentiate MSCs

**Figure 1:**
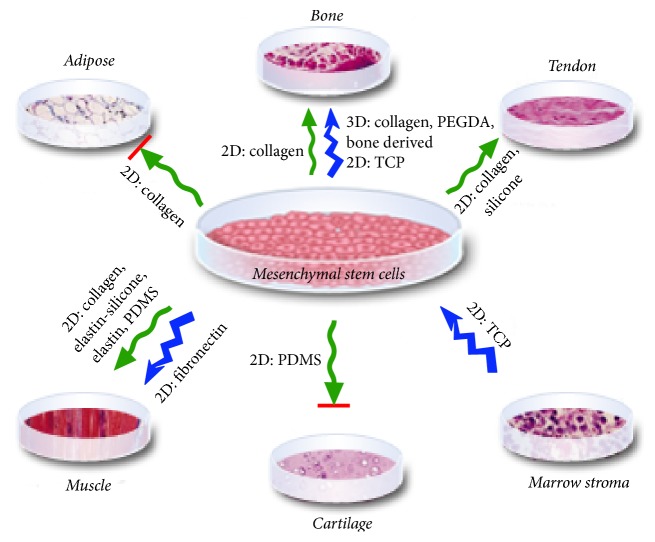
Diagram representing the effects of vibration (blue jagged arrow) and cyclic tensile strain (green squiggly arrow) on MSCs. The arrows depict the loading type. The italics detail the* in vitro* culture conditions in which the differentiation into the indicated lineage was observed. Red lines indicate inhibition of the downstream lineage. Tissue images from Tuan et al. [4], CC BY 4.0.

When maintained* in vitro*, MSCs can be chemically and mechanically differentiated into a variety of tissues such as bone, cartilage, tendon, and ligament [2]. The biochemical factors that promote specific cell responses are well understood and thus enable researchers to successfully guide cell differentiation. Adding chemical factors to cell culture media such as ascorbate, dexamethasone (dex), and bone morphogenetic proteins (BMPs) promotes osteogenesis; serum-free medium and transforming growth factor-*β*_1_ (TGF-*β*_1_) promote chondrogenesis; growth and differentiation factor 5 (GDF-5) promotes tenogenesis; platelet derived growth factor (PDGF) promotes myogenesis; and dex, insulin, and 3-isobutyl-1-methylxanthine (IBMX) promote adipogenesis (Figure 1) [3–5]. In addition to chemical factors, mechanical properties such as scaffold stiffness can be used to guide MSC differentiation [1, 6]. For instance, dex is widely used to promote MSC osteodifferentiation and alternately scaffolds with elastic moduli comparable to bone promote MSC osteodifferentiation [3, 6].

In their native environment tissues are subjected to a variety of biochemical signals in addition to a multitude of loading conditions that influence their development. In the absence of appropriate biochemical factors or scaffold mechanical properties, appropriate loading can drive MSC differentiation towards a desired fate [7]. Conversely, inappropriate loading can inhibit a desired fate [8]. MSC response to loading is dependent on stress/strain magnitude, duration, loading type, and force propagation through cytoskeletal configuration and attachment site geometry (for a recent review, see Delaine-Smith and Reilly) [3]. Loading types include such parameters as tension, compression, shear, bending, torsion, electromagnetic inputs, and vibration. Furthermore, loading can be separated into static or cyclic loading. All these loading types are experienced in situ by cells and often in combination. For example, in bone marrow, tension, compression, and fluid-induced shear may all be present but the effects of these forces on the stem cells within the bone marrow are not well understood [3, 9]. A challenge of tissue engineering is identifying the appropriate combination of chemical and mechanical parameters that will differentiate harvested MSCs into specific cell types* in vitro*. Although chemical inducers alone can drive differentiation* in vitro*, after scaffold implantation any biofactors it contains will eventually dissipate; thus the success of the scaffold will be maximized if its mechanical properties continue to influence cells.

## 3. The Effect of Vibration on Mesenchymal Stem Cells

Although vibration is not necessarily a loading condition experienced in nature, an extensive number of* in vivo* studies have been conducted with whole body vibration [33–39]. Whole body vibration studies have been used to model the cyclic tensile strain imparted on muscle or bone during physical actives such as walking, stair climbing, or weight lifting exercises [34]. The vibration stimulates the skeleton in a manner similar to walking or running and has been found to increase bone mass and bone strength [33–35, 40]. Whole body vibration stimulates osteogenesis of MSCs through mechanotransduction, resulting in a bone mass increase [35, 39]. Whole body vibration may also elicit a response from differentiated cells, which influence MSC differentiation [35]. Investigations exploring the effect of whole body vibration on MSCs may be confounded by the concurrent effect of vibration on differentiated cells. The mechanism of mechanotransduction during vibration in MSCs and subsequent response is not fully understood [36–38]. Additionally, the in situ response of cells to an external load cannot be separated from the systemic response of the whole organism.

Thus, researchers also explore* in vitro* parallels to whole body vibration to tease out the response of MSCs to vibration (summarized in Table 1). As such, the cell environment and loading factors can be controlled. When subjecting MSCs to vibration, investigators specifically select loading parameters, biochemical additives, and cellular environment to observe or induce differentiation of cells.

### 3.1. Tissue Culture Plastic

Most* in vitro* vibration studies are performed with a cell monolayer cultured on tissue culture plastic (TCP) [10–13, 41]. The MSC response to vibration depends on frequency, acceleration, and duration of stimulation.

**Figure 2:**
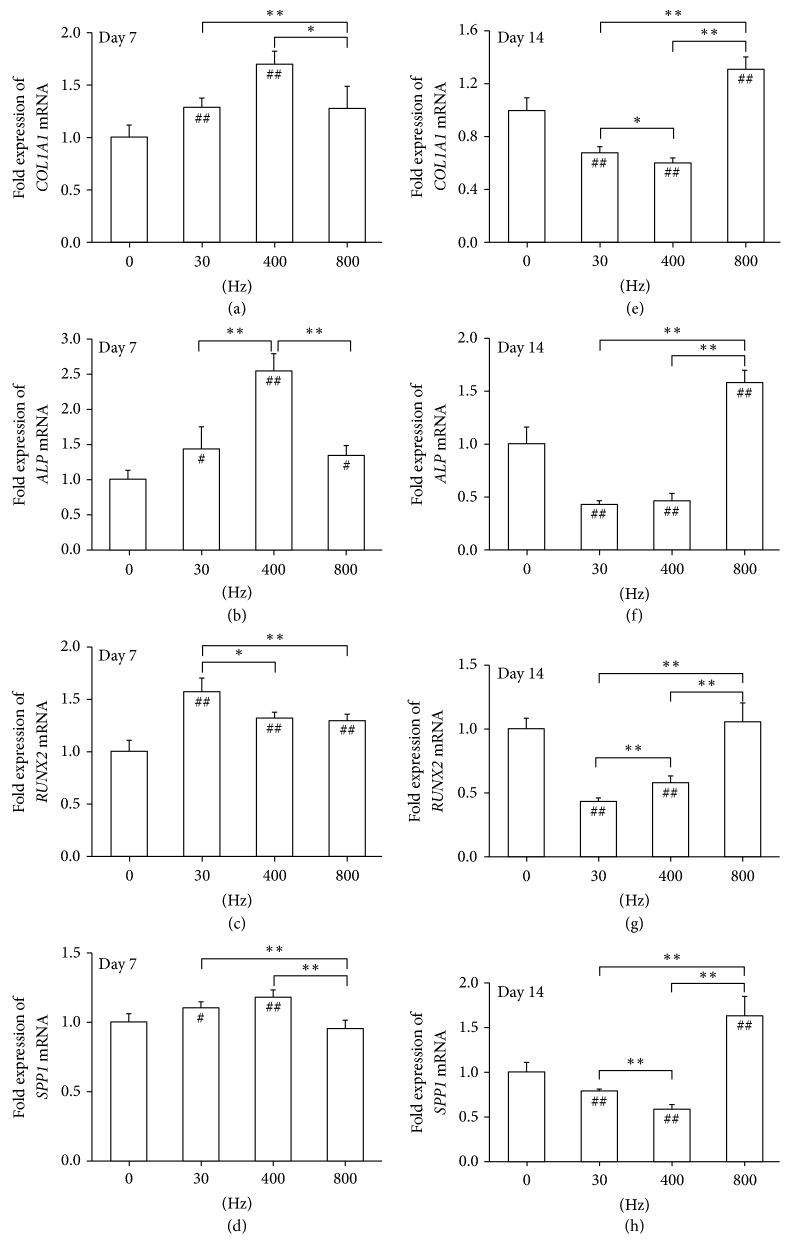
Acoustic-frequency vibratory stimulation (AFVS) modulates expression of mRNA encoding osteogenesis-specific markers in human bone marrow-derived mesenchymal stem cells (BM-MSCs) at the time points of day 7 ((a), (b), (c), and (d)) and day 14 ((e), (f), (g), and (h)). The mRNA levels of COL1A1 ((a), (e)), ALP ((b), (f)), RUNX2 ((c), (g)), and SPP1 ((d), (h)) were measured by real-time RT-PCR. Values are mean ± standard error of four independent experiments (*n* = 4). ^*∗*^*P* < 0.05; ^*∗∗*^*P* < 0.01 in the indicated groups from unpaired* t*-test. ^#^*P* < 0.01; ^##^*P* < 0.01 compared with the 0 Hz control group from unpaired* t*-test. From Chen et al. [10], CC BY 3.0.

Chen et al. investigated the effects of 0.3 g acoustic vibration at 30, 400, and 800 Hz on human MSCs (hMSCs) in cell culture plates [10]. Cells were stimulated 30 min/day for 7 days. The investigators selected 30 Hz because they noted that osteogenesis was promoted following whole body vibration under 100 Hz [35] and they selected higher frequencies because higher frequencies are more suited for localized body vibrations [10]. The authors found that cell proliferation, calcium deposition, and collagen 1 (Col I) gene expression increased the most following 800 Hz vibrations at 0.3 g (Figure 2). At 800 Hz, adipogenic gene expression and lipid accumulation were decreased while at 30 Hz adipogenesis was promoted. Though acoustic vibration differs from direct mechanical vibration, both methods impart physical vibration to the cells.

Demiray and Özçivici cultured mouse MSCs on glass cover slides within 6 well plates [11]. Plates were stimulated with low magnitude (<1 g) and high frequency (20–90 Hz; LMHF) vibrations of 90 Hz at 0.15 g over 7 days for 15 min/day. The authors hypothesized that low intensity vibrations would induce MSC differentiation into osteogenic cells. Following vibration, cells were tested for osteogenic markers Runx2 and osteocalcin (OC) to identify osteogenic differentiation. While gene expression of vibrated and control cells was similar, the vibrated cells exhibited increased proliferation and morphological changes. Vibrated cells also displayed increased cellular height and increased molecular expression of focal adhesion kinase.

Lau et al. studied the effects of LMHF vibration on rat MSCs cultured on TCP while using osteogenic media [12]. Cells were stimulated with 60 Hz vibrations at 0.3 g for six 1-hour bouts. The authors hypothesized that the vibration would promote osteogenesis based on prior animal and human studies [35, 42]. Following vibration, cells were tested for osteoblast-specific transcription factor Osterix (Osx) to detect osteoblastic differentiation. The MSCs displayed decreased Osx levels and inhibited mineralization, indicating that LMHF vibration did not enhance osteogenic differentiation but seemed to inhibit it. Further, LMHF vibration did not affect proliferation rate. As both the control and test groups contained osteogenic media, rather than a true investigation of the effects of LMHF, the study was more an investigation of the combined effect of LMHF and osteogenic media compared to osteogenic media alone.

Kim et al. tested hMSCs with a wide array of vibration frequencies and accelerations [13]. MSCs were seeded on TCP or a collagen sponge. The collagen sponge was prepared from a cross reaction of chondroitin-6-sulfate and type I collagen [43]. The cells were seeded within the pores of the sponge, creating a multidimensional scaffold. Cells were subjected to varying accelerations of vertical vibration for 10 min/day for 5 days using a custom platform on a shaker. Accelerations varied between 0.1, 0.2, 0.3, 0.4, 0.5, and 0.6 g and frequencies varied between 10, 20, 30, and 40 Hz. Vibration on TCP resulted in a minor increase of proliferation. At 0.2 g and 0.3 g accelerations, proliferation rates increased as frequency increased. For all frequencies, proliferation was significantly higher at 0.3 g compared to other accelerations. The highest proliferation was observed at 0.3 g for both 30 Hz and 40 Hz. Thus, their subsequent experiments were performed with 30 Hz vibrations delivering 0.3 g accelerations. In their differentiation assays, the authors found that the osteoblastic differentiation markers, alkaline phosphatase (ALP) and osteopontin (OPN), were upregulated in vibrated cells while the osteoblastic markers, OC and bone sialoprotein (BSP), were unaffected. Alizarin red staining was increased in MSC monolayers receiving vibration and osteogenic media compared to control, though staining was not increased for vibrated cells that did not receive osteogenic media. MSCs behaved differently on scaffolds compared to TCP. Specifically, while OPG, Col I, and VEGF expression showed significant increases in vibrated groups compared to nonvibrated groups, this effect was observed only in MSCs cultured on scaffolds. These differing results suggest that additional factors, such as microarchitectural differences between scaffolds and TCP, may influence the mechanotransduction of vibration.

It is well known that cells in culture do not behave the same on TCP as they do in scaffolds or in their natural environments [44–48]. The same holds true for cells subjected to vibration [12, 13, 21, 43, 44]. Further, vibrated cells on TCP do not necessarily behave identically between similar investigations. In several LMHF studies, osteogenesis was increased significantly when MSCs on TCP were vibrated at accelerations of 0.3 g at 35 Hz or 45 Hz [49, 50]. Conversely, in other studies that vibrated MSCs on TCP with 0.3 g accelerations at 30 Hz or 60 Hz, osteogenesis was inhibited [13, 21]. Considering the variation in MSC response that TCP elicits, scaffolds may provide a more accurate and consistent in situ representation of the MSC response to vibration.

### 3.2. Two-Dimensional Scaffolds

Scaffolds provide a more complex cellular interaction than a simple monolayer on TCP. A 2D scaffold has cells cultured on a flat surface while a 3D scaffold has cells embedded within or seeded on a multidimensional surface. Two-dimensional scaffolds for vibration studies are often membranes coated with osteogenic proteins or minerals [15, 16, 51]. Cell attachment and force transmission vary with different scaffolds or bound matrix proteins.

Edwards and Reilly seeded hMSCs in gelatin coated 12-well plates [14]. Plates were subjected to LMHF vibrations of 15, 30, 45, or 60 Hz at 0.02 g. Vibration occurred for 10 or 45 minutes. The authors also investigated the effect of osteogenic media (media containing dex) during their vibration studies. Alkaline phosphatase (ALP) activity was the only measurement of cell commitment. ALP activity was greatest following 45-minute vibrations of 60 Hz with osteogenic media. Forty-eight hours after stimulation, MSCs subjected only to vibration did not demonstrate a statistically relevant increase of ALP activity. ALP activity was greater in cells that had dex+ media compared to dex− media. For dex+ cells, 60 Hz stimulation had a statistically greater ALP activity compared to other frequencies.

Sen et al. investigated the inhibition of adipogenesis in mouse MSCs after vibration [15]. The authors subjected MSCs seeded on a collagen-coated silicon membrane to low intensity vibrations of <10 microstrain, at 90 Hz. The MSCs were stimulated for 20 minutes twice a day. Following vibration, cells were tested for the adipogenic markers adiponectin, PPAR*γ*2, and aP2. The MSCs expressed decreased levels for all adipogenic markers and the development of lipid granules was inhibited. The authors also investigated the synergistic effect of vibration and adipogenic media. The MSCs subjected to vibration and adipogenic media had a statistically insignificant expression of adipogenic markers. The results indicate that adipogenesis was inhibited in cells subjected to vibration and that treatment with adipogenic media did not recover adipogenesis. Conversely, markers for osteogenic differentiation were promoted significantly after vibration, though this effect was only observed in MSCs treated with adipogenic media.

Tong et al. subjected hMSCs to 200 Hz acoustic vibration to replicate vocal cord vibrations [16]. Cells were seeded on PCL scaffolds coated with fibronectin and subjected to vibrations for 12 hrs/day over 7 days continuously or discontinuously. All vibrated cells expressed enhanced F-actin and a5b1 integrin expression. Levels of vocal fold extracellular matrix components were significantly elevated. Myogenic differentiation in MSCs were indicated by elevated levels of tenascin-C, collagen III, and procollagen I, while osteogenic markers were not expressed.

Edwards and Reilly, similar to Lau et al., found that a synergist effect of vibration and osteogenic media promoted osteogenesis [12, 14]. Sen et al. found adipogenesis to be inhibited after vibration, even with the addition of adipogenic media. Tong et al. found myogenic differentiation of hMSCs seeded on fibronectin-coated scaffolds [16]. Each of these studies used a different biological coating on a synthetic scaffold. It is important to consider that the different MSC responses to vibration observed by these studies may in part be explained by different MSC responses to the scaffolds. In short, the biological components of the scaffolds are potentially introducing a compounding biological variable to the investigations, contributing to the observed synergistic responses.

### 3.3. Three-Dimensional Scaffolds

Few studies of MSC response to vibration* in vitro* have used three-dimensional substrates [10–13, 15, 16, 51]. Three-dimensional substrates translate mechanical force to cells via different mechanisms than 2D substrates [52]. Three-dimensional substrates may better model in situ cell attachment and the resultant effects of mechanical loading [53]. In 3D environments, there are increased cell-to-cell contact and cell-to-extracellular matrix interactions compared to 2D monolayers [53]. Due to these factors, cells within 3D scaffolds likely better model the* in vivo* response to vibration than cells on 2D substrates.

#### 3.3.1. Natural Scaffolds

Kim et al. vibrated hMSCs after inoculation on a collagen sponge [13]. Cells were exposed to 30 Hz vibration at 0.3 g. In their differentiation assay, the authors found that the osteogenic markers OPG, Col I, and VEGF expression were increased after MSCs were vibrated. These results differ from the previously described results of Chen et al., who found increased adipogenesis in TCP-monolayer hMSCs subjected to 30 Hz vibration at 0.3 g. The difference in the response of MSCs within a 3D scaffold and MSCs in a monolayer suggests that factors such as microarchitectural cues may mechanotransduce vibration.

**Figure 3:**
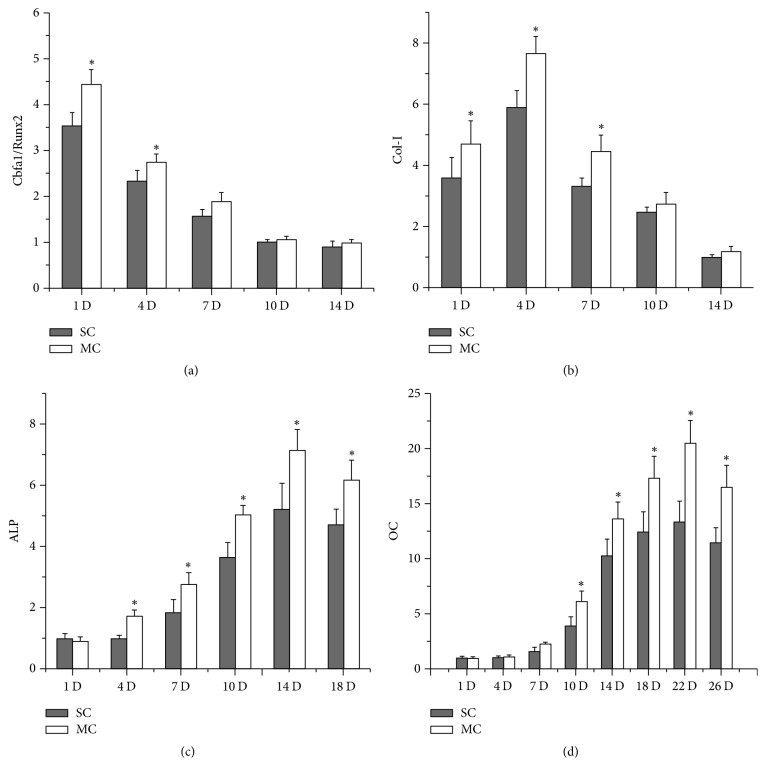
Effect of microvibration on osteogenic gene expressions in BMSC cellular scaffolds. Cbfa1/Runx2, Col I, ALP, and OC mRNA expressions were assayed on days 1, 4, 7, 10, 14, 18, 22, and 26. Data show that microvibration greatly upregulated these mRNA levels at different stages of osteogenesis. Each bar represents the mean ± standard deviation (*n* = 3); ^*∗*^*P* < 0.05. SC, static culture. MC, microvibration culture. Col I, collagen I. ALP, alkaline phosphatase. OC, osteocalcin. From Zhou et al., [17] with kind permission from eCM journal (http://www.ecmjournal.org).

Zhou et al. subjected rat MSCs seeded on 3D bone-derived scaffolds to LMHF vibration [17]. The hollow components of the scaffolds allowed cells to attach within the multidimensional matrix. Rat MSCS seeded on TCP were used as control groups. The authors vibrated cells with 40 Hz at 0.3 g for 6 hours. The vibrated MSCs demonstrated increased levels of osteogenic markers ALP, Coll I, and OC. ALP activity was significantly higher in cells vibrated within 3D scaffolds than cells vibrated on TCP (Figure 3). However, vibration resulted in lower proliferation after day 7. The increased response from vibrated MSCs within 3D scaffolds compared to MSCs on TCP should motivate further investigation of MSCs within 3D scaffolds.

#### 3.3.2. Synthetic Scaffolds

To the author's knowledge, our laboratory has conducted the only vibration studies on MSCs entrapped within a synthetic material [18]. Cells entrapped within PEGDA microspheres were subjected to vibrations of 100 Hz at 0.3 g, 3.0 g, or 6.0 g for 24 hours. Cells were subsequently tested for adipocyte, chondrocyte, and osteoblast differentiation. Osteogenic differentiation in MSCs was observed at 0.3 and 3.0 g accelerations, while 6.0 g accelerations were lethal to cells. Alkaline phosphatase activity was observed on day 4 in MSCs subjected to 0.3 and 3.0 g. Alizarin red staining was also significantly increased in 0.3 and 3.0 g MSCs compared to nonvibrated controls. Chondrogenesis and adipogenesis were not observed at any time point.

The vibration studies expose MSCs to a range of accelerations and frequencies. Accelerations from 0.02 g to 6.0 g were used in combination with frequencies ranging from 10 to 800 Hz (Table 1). With such variations in vibration parameters, it is unsurprising that there is a great variation in observed response. However, when probing further, the disparity in cell response is mostly observed in cells seeded on 2D scaffolds. MSCs on or within 3D scaffolds uniformly differentiated to bone. While 3D scaffolds were tested in a smaller range of vibration parameters (0.3–6 g; 30–100 Hz), these studies may point to a uniform response to MSCs when vibrated within a 3D environment.

## 4. The Effect of Cyclic Tensile Strain on MSCs

Cyclic uniaxial tensile strain can be applied to cells encapsulated in or seeded on a flexible scaffold. Rigid materials such as TCP cannot be used for tensile strain studies. Silicon scaffolds are regularly used as a synthetic, flexible scaffold. The effects of tensile strain on MSCs, inducing tenogenic, osteogenic, and myogenic responses, have been investigated and are reviewed below [3, 19–32].

### 4.1. Two-Dimensional Scaffolds

#### 4.1.1. Natural Scaffolds

The following studies investigate the effects of cyclic uniaxial tensile strain with magnitudes between 0.8% and 15% at 1 Hz on MSCs seeded on collagen membranes and other scaffolds (Table 2) [19–22]. The strain magnitudes were selected by the authors to replicate the tensile strain experienced in situ by bone, muscle, and tendon.

Chen et al. seeded hMSCs on collagen type I coated scaffolds and then subjected them to 3% and 10% cyclic tensile strain at 1 Hz for 8 or 48 hours [21]. The authors were investigating osteogenic or tenogenic commitment of MSCs following such strain. For all strains, organized cell alignment was noted. Cells subjected to both strain rates became longer, slenderer in shape, and oriented perpendicular to the axis of strain. hMSCs subjected to 3% strain for 8 hours demonstrated an upregulation of osteoblastic markers with increased levels of ALP and Cbfa1. hMSCs strained at 10% for 48 hours demonstrated significant increases in type I collagen, type III collagen, and tenascin-C, indicating tenogenic differentiation. The authors suggested strain amplitude and duration of strain may influence tenogenic and osteogenic commitment of MSCs.

**Figure 4:**
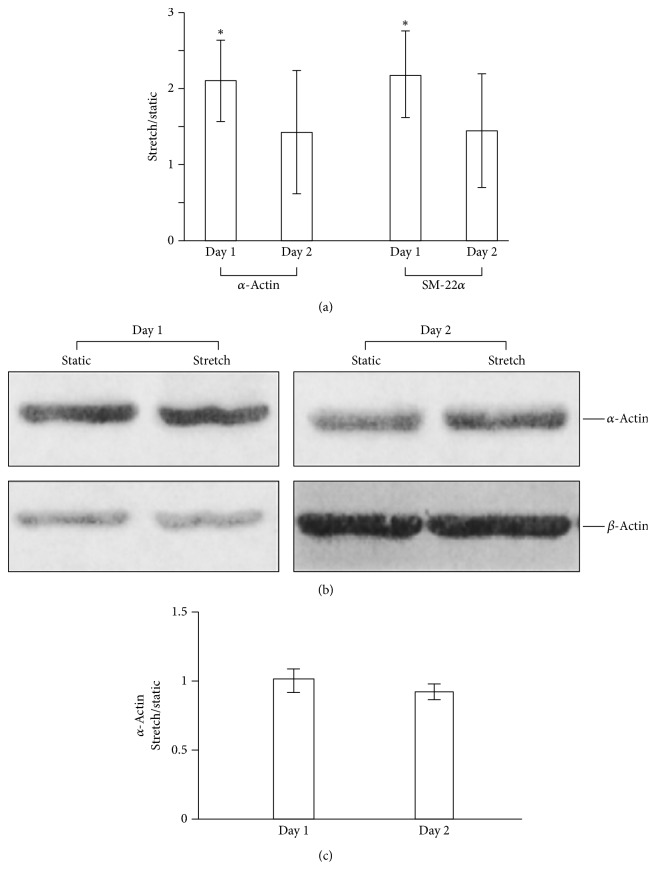
Effects of uniaxial strain on SM marker expression in MSCs. MSCs were cultured on collagen-coated elastic membranes for 1 day and subjected to 10% uniaxial strain at 1 Hz or kept as static controls for 1 and 2 days. (a) The RNA from each sample was reverse-transcribed into cDNA and the gene expression of SM a-actin, SM-22a, and GAPDH was analyzed by qPCR with their respective primers. The expression level of each gene was normalized with the level of GAPDH in the same sample. The ratio of the gene expression (stretch/static) is presented as mean (±) standard deviation from at least three experiments. ^*∗*^Significant difference (*P* < 0.05) from 1. (b) The protein expression of SM a-actin and actin was analyzed by immunoblotting with respective antibody. (c) Statistical analysis of protein expression. The protein expression was quantified, and the expression level of a-actin was normalized with the level of h-actin in the same samples. The ratio of the normalized protein expression (stretch/static) is presented as mean (±) standard deviation from at least three experiments. From Park et al., Biotechnology and Bioengineering [20]. Copyright© 2004 by John Wiley Sons, Inc. Reprinted by permission of John Wiley & Sons, Inc.

Park et al. aimed to replicate the strain conditions of vascular smooth muscle on hMSCs seeded on elastin or collagen-coated membranes [20]. MSCs were subjected to 10% uniaxial tensile strain at 1 Hz for 24 hours. Smooth muscle cell (SMC) and osteogenic markers were investigated. Following strain, cells in both scaffolds increased collagen I expression; however, markers of osteogenic differentiation were not significant. After being subjected to strain, levels of smooth muscle markers *α*-actin, SM-22*α*, and *β*-actin were transiently increased in cells on both scaffolds (Figure 4). Expression of *α*-actin and SM-22*α* subsequently decreased shortly after cells aligned themselves perpendicular to the direction of strain. After 3 days, SM-22*α* decreased by 50% on collagen-coated scaffolds and 25% on elastin-coated membranes. Levels of Col I also decreased after alignment. The authors concluded that uniaxial strain may promote MSC differentiation into SMCs if the cell orientation is fixed. Interestingly, the decrease in gene expression after alignment described by Park et al. contradicts the stable gene expression described by Chen et al., who did not observe decreased gene expression from MSCs subjected to 10% strain [20, 21].

Khani et al. investigated the mechanical properties of hMSCs subjected to uniaxial strain with or without chondrogenic media (with TGF-*β*_1_) [19]. hMSCs were seeded on poly(dimethyl siloxane) (PDMS) with a collagen coating to enable cell attachment. For 24 hours seeded hMSCs were subjected to uniaxial strain of 5% at 1 Hz, comparable to physiological levels within human arteries. Strained cells without TGF-*β*_1_ had significantly increased Young's Moduli (*E*) and elevated levels of the smooth muscle markers ASMA, h1-Calponin, and SM22A. The strained hMSCs demonstrated increased myogenesis with or without TGF-*β*_1_ in the media. After stimulation, these cells had also become aligned perpendicular to the axis of strain. The authors suggested that the realignment of these cells may reinforce the material, creating a stiffer composition, and may ultimately impact mechanotransduction.

Koike et al. subjected hMSCs seeded on collagen I coated membranes to 0.8%, 5%, 10%, and 15% cyclic strain at 1 Hz for 2 days [22]. Cell proliferation significantly increased at 5%, 10%, and 15% strain compared to unloaded controls. At 1-hour and 6-hour markers, Cbfa1/Runx2 increased at 0.8% and 5% strain but decreased at 15% strain. At 24 hours and 48 hours, cell proliferation and Col I increased at 5%, 10%, and 15% strain while Cbfa1/Runx2 expression, osteocalcin expression, and ALP activity were significantly decreased. ALP activity was increased at 0.8% strain. These results indicate that high magnitude mechanical strain will inhibit osteoblastic differentiation, while strain at low magnitudes may enhance osteoblastic differentiation.

All studies described above used collagen scaffolds to investigate the response of hMSCs to uniaxial tensile strain [19–22]. On collagen scaffolds, MSCs subjected to low magnitude tensile strains (≤3%) underwent osteogenic differentiation [21, 22, 54]. At greater tensile strains (5%), myogenic differentiation was promoted [19, 22]. hMSCs subjected to high magnitude tensile strains (≥10%) showed an inhibition of osteogenic differentiation, transiently enhanced myogenic differentiation, and enhanced tenogenic differentiation [20–22]. The literature generally agrees that MSC osteodifferentiation occurs at lower strain magnitudes than MSC tenodifferentiation [19–22, 54].

#### 4.1.2. Synthetic Scaffolds

Park et al. noted a difference in MSC response to a synthetic scaffold compared to a collagen scaffold [20]. As described in Section  4.1.1, Park et al. subjected MSCs seeded on elastin-coated or collagen-coated scaffolds to 10% uniaxial tensile strain at 1 Hz for 24 hours. The hMSCs transiently upregulated smooth muscle markers. Smooth muscle gene expression was higher in cells seeded on the elastin scaffolds. The authors suggested that the MSCs sensed a difference in the mechanical loading of the two microenvironments.

Zhang et al. subjected rMSCs seeded on a silicone scaffold to 10% tensile strain at 1 Hz [23]. The authors investigated the effect of tensile strain on tenogenesis of hMSCs. The authors also investigated the effects of coculturing hMSCs with ligament fibroblasts. Only cells subjected to strain exhibited morphological changes. After tensile strain, rMSCs had a more elongated fibroblast-like cell type. The strain triggered an early upregulation of Col I and Col III. Tenascin-C expression was also upregulated in cells subjected to strain. Cells subjected to strain demonstrated greater levels of tenogenic gene expression than cells cocultured with fibroblasts but not subjected to strain.

Huang et al. subjected rMSCs seeded on an elastic-silicone membrane to 5%, 10%, 15%, and 20% tensile strain at 1 Hz for 24 hours [24]. The authors investigated the presence of cardiac-related gene expression using negative controls and positive controls. Cells subjected to cyclic strain expressed GATA-4, *β*-MHC, NKx2.5, and MEF2c. Gene expression was greatest in cells subjected to 10% strain. The researchers then subjected rMSCs to 10% strain at 1 Hz for 24, 48, and 72 hours. The expression of GATA-4, *β*-MHC, NKx2.5, and MEF2c was significantly increased for all durations of strain. The investigators suggested that cyclic mechanical strain of 10% at 1 Hz induces cardiomyogenic differentiation of MSCs.

Jagodzinski et al. applied tensile strain to hMSCs seeded on a silicone scaffold [55]. Cells were subjected to 2% or 8% strain, six hr/day for three days at 1 Hz. hMSCs were cultivated with (dex+) or without (dex−) dexamethasone. For both strain magnitudes, cells had significantly increased ALP secretion and collagen III upregulation. Cells subjected to 8% strain significantly upregulated Col I and Cbfa1. Cells that underwent strain had significantly greater gene expression, with or without dex, for all markers of gene expression. At both high and low magnitudes of cyclic strain, hMSC osteogenic commitment was enhanced on silicon scaffolds, contrary to the observed trends of hMSCs on collagen scaffolds, which showed tenogenic commitment at high strain magnitudes and osteogenic commitment at low strain magnitudes.

After being subjected to 10% strain, MSCs seeded on synthetic scaffolds demonstrated enhanced myogenic differentiation, resulting in fibroblasts, smooth muscle cells, and cardiac cells [20, 23, 43]. For other strain magnitudes, MSCs seeded on synthetic scaffolds respond differently to tensile strain than MSCs seed on natural scaffolds [20, 23, 24, 55]. Cells on synthetic scaffolds had a greater expression of smooth muscle markers compared to cells on natural scaffolds [20]. Osteodifferentiation was induced at high magnitudes of strain in cells on silicone while osteogenesis was inhibited at high magnitudes of strain in cells on collagen [55]. Thus, demonstrating that the MSC response to strain differs on synthetic-based scaffolds compared to natural scaffolds.

While the preceding scaffolds were comprised of synthetic materials, only one was not modified with elastin. The MSCs on scaffolds comprised of or modified with elastin all exhibited myodifferentiation. The MSCs on truly synthetic scaffolds exhibited tenodifferentiation. Thus, for 2D scaffolds, scaffold type may be more important than the loading.

### 4.2. Three-Dimensional Scaffolds

Cells entrapped within a scaffold or seeded throughout a structure are subjected to a different microenvironment than cells seeded on a planar scaffold. Subtle differences in loading or cell-cell communication may impact cell response to strain. The following studies investigate the effects of cyclic strain on cells entrapped within 3D scaffolds (Table 3).

#### 4.2.1. Natural Scaffolds

The first study to investigate hMSCs entrapped in 3D collagen matrix under cyclic strain was conducted by Sumanasinghe et al. [25]. The authors subjected hMSCs entrapped within a collagen matrix to 10% or 12% uniaxial cyclic tensile strain at 1 Hz for 4 hr/day. Strain was applied for 7 or 14 days. hMSCs remained highly viable for all strain conditions. hMSCs subjected to 10% strain demonstrated a significant increase in BMP-2 expression for both durations of strain. Cyclic strain of 12% induced a significant increase in BMP-2 expression only in cells subjected to 14 days of strain. The authors concluded that strain alone can induce osteogenic differentiation without the addition of osteogenic supplements.

Sumanasinghe et al. conduced a follow-up study to investigate the expression of proinflammatory MSC cytokines using identical strain conditions (10 or 12%; 1 Hz for 4 hr/day; 7 or 14 days) [26]. The authors also used osteogenic media to evaluate the combined effect of cyclic strain and osteogenic supplements. Initially, hMSCs undergoing strain had reduced viability. After day 6, hMSCs subjected to 10% strain had increased viability. Only strained cells receiving osteogenic media had increased levels of TNF*α* and IL-I*β*. The authors demonstrated that hMSCs entrapped within a collagen matrix maintain high viability after cyclic strain.

Charoenpanich et al. entrapped hMSCs, specifically adipose derived stem cells, within a collagen I gel sheet [56]. The entrapped cells were subjected to 10% cyclic tensile at 1 Hz for 4 hr/day over 14 days. The authors performed a microarray analysis of 847 genes and found 184 transcripts affected by tensile strain. Network analysis suggested that strain may impact osteogenic differentiation by upregulation of proinflammatory cytokine regulator interleukin-1 receptor antagonist (IL1RN) and angiogenic inductors including fibroblast growth factor 2 (FGF-2) and vascular endothelial growth factor A (VEGF-A). Cells subjected to strain and osteogenic media resulted in significantly increased calcium deposits, suggesting a synergistic effect of the strain and media driving the cells towards osteogenic differentiation.

Qiu et al. applied cyclic strain to hMSCs seeded along collagen fibers to investigate fibroblastic differentiation [27]. The fibrous scaffold provided a nonplanar microenvironment. The hMSCs were subjected to 10% tensile strain at 1 Hz for 12 hrs/day over 14 days. Collagen I, collagen III, tenascin-C, and fibroblastic transcription factor scleraxis were all found to be significantly upregulated in cyclically strained hMSCs compared to unstrained control cells. Thus, 10% cyclic strain significantly promoted tenogenic differentiation of hMSCs.

Juncosa-Melvin et al. applied strain to rabbit MSCs seeded within collagen sponges [28]. MSCs were subjected to cyclic strain of 2.4% at 0.003 Hz for 8 hrs/day over 12 days. Tenogenic differentiation was not conclusively promoted by cyclic strain, but significant gene expression of collagen I and collagen III was induced by cyclic strain. Strained MSCs showed 3 or 4 times greater collagen I and collagen III production compared to unstrained controls. However, gene expression of fibronectin or decorin was not significantly increased in strained MSCs.

Few studies investigate both the effects of 3D scaffolds and cyclic tensile strain on hMSC differentiation without osteogenic supplements. In the few studies that do not use osteogenic media, most use scaffolds or coatings comprised of collagen, which is a major component in bone and hence provides a biological factor that induces its own cell response [57, 58].

#### 4.2.2. Synthetic Scaffolds

Rathbone et al. investigated the response of hMSCs to cyclic tensile strain entrapped within 3D hydrogels with either the cell attachment tripeptide, arginylglycylaspartic acid (RGD), or a dummy tripeptide, arginyl-glycyl-glutamic acid (RGE) [29]. Cells were either entrapped within a hydrogel with cell attachment sites (RGD) or entrapped within a hydrogel without cell attachment sites (RGE). The authors' hydrogel was composed of Fmoc-FF:Fmoc-RGD/RGE. The hMSCs were subject to 3% strain 1 Hz for 1 hour or 24 hours and evaluated 2 hours or 24 hours after strain. Cells within hydrogels demonstrated high viability. The authors investigated CCNL2, WDR61, and BAHCC1 as potentially important mechanosensitive genes. After 1 hour of strain, hMSCs on monolayers significantly downregulated CCNL2, WDR61, and BAHCC1. After 24 hours of strain, hMSCs on monolayers significantly upregulated BAHCC1. BAHCC1 was not expressed by hMSCs in either of the 3D scaffolds. WDR61 was significantly upregulated by hMSCs in both 3D scaffolds after 1 hr of strain. CCNL2 was upregulated in hMSCs only in scaffolds with RGD. The cell response differed when in a monolayer or in a 3D scaffold and when within 3D scaffolds with or without RGD, thus indicating the impact of attachment sites on mechanotransduction in otherwise identical scaffolds.

Kreja et al. applied strain to hMSCs seeded throughout a novel textured PLA scaffold to investigate fibroblastic differentiation [30]. Cells were subjected to 2% or 5% strain at 1 Hz for 1 hr/day over 15 days. The authors analyzed the gene expression of ligament matrix markers: collagen I, collagen III, fibronectin, tenascin-C, decorin, MMP-1, MMP-2, and inhibitors TIMP-1 and TIMP-2. Cells subjected to strain did not demonstrate significant gene expression except in the downregulation of both MMP-1 and TIMP-2 in cells subjected to 5% strain. For both strain parameters, tenogenic differentiation was not promoted in hMSCs.

Yang et al. investigated the effects of strain on hMSCs entrapped within fast and slow degrading MMP-sensitive PEG hydrogels to investigate tenogenic differentiation [31]. Cells were subjected to 10% strain at 1 Hz for 12 hrs/day over 14 days. Cell realignment in response to the strain direction was not observed. hMSCs within the slow degrading hydrogel upregulated collagen III by 3.8-fold and upregulated tenascin-C by 2.5-fold while hMSCs within the fast degrading hydrogel upregulated collagen III by 2.1-fold and upregulated tenascin by 1.7-fold. The authors suggested that cyclic straining promoted tenogenic differentiation and that the presence of strain had a greater influence on cell differentiation than the difference in composition between the hydrogels.

Doroski et al. expanded the Yang et al. investigation where hMSCs entrapped with PEG-based hydrogels were cyclically strained [31, 32]. The researchers entrapped hMSCs within oligo(poly(ethylene glycol) fumarate) (OPF). Cells were then subjected to 10% strain at 1 Hz for 12 hrs/day over 21 days. By day 21, cyclic strain significantly upregulated the tenogenic markers collagen I, collagen III, and tenascin-C, while osteogenic, chondrogenic, and adipogenic markers were not increased. Thus, the cyclic strain promoted tenogenic differentiation.

It is interesting to note that collagen derived scaffolds mostly resulted in osteodifferentiation while synthetic scaffolds mostly resulted in tenodifferentiation. These results indicate that if seeking to differentiate MSCs using mechanical loading such as tensile strain, the scaffold material type will influence the MSC fate as much as the loading parameters will.

## 5. Summary

The response of MSCs subjected to cyclic strain and vibration appear to vary with loading parameter as much as it varies with culture conditions. Unsurprisingly, for similar loading conditions, TCP produced different results than 2D scaffolds, which in turn produced different results than 3D scaffolds. The varying effects of natural and synthetic scaffolds on MSC differentiation may be explained by the differing elastic moduli between these scaffolds or it may be the distinct microarchitecture of a natural scaffold compared to featureless synthetic scaffolds, or the dominating factor may be the native biochemical cues contained within natural scaffolds. These questions warrant further investigation if mechanical loading is to be pursued as an alternate method to induce MSC differentiation.

## 6. Future Perspectives

Future investigations of MSC differentiation using vibration and cyclic strain should explore varying types of natural scaffolds in concert with the loading parameters. Collagen is a primary component of bone and as such makes an ideal bone scaffold. Vibration within 3D collagen scaffolds ultimately led to osteogenic differentiation. Future studies utilizing scaffolds optimized for myogenic, adipogenic, or tenogenic differentiation should be explored to tease out whether vibration and/or cyclic strain in 3D scaffolds consistently leads to osteogenic differentiation or whether vibration and/or cyclic strain in 3D scaffolds leads to tissue optimized for that 3D scaffold.

## Figures and Tables

**Table 1:** Effect of vibration on MSCs.

Environment		Cell	Acceleration	Frequency [Hz]	Results	Ref.
TCP		hMSC	0.3 g	30, 400, 800	Osteogenic	[10]
		mMSC	0.15 g	90	Osteogenic	[11]
		rMSC	0.3 g	60	Osteogenesis inhibited^*∗*^	[12]
		hMSC	0.1–0.6 g	10, 20, 30, 40	Cell proliferation	[13]

2D	Gelatin	hMSC	0.02 g	15, 30, 45, 60	Inconclusive^*∗*^	[14]
	Collagen	mMSC	10 *µ*strain	90	Adipogenesis inhibited	[15]
	Fibronectin	hMSC	Acoustic	200	Myogenic	[16]

3D	Collagen Sponge	hMSC	0.3 g	30	Osteogenic	[13]
	Bone derived	rMSC	0.3 g	40	Osteogenic	[17]
	PEGDA	hMSC	0.3, 3, 6 g	100	Osteogenic	[18]

*∗* indicates the addition of differentiation media.

**Table 2:** Effect of cyclic tensile strain on MSCs seeded on 2D scaffolds.

Scaffold		Cell	Strain (%)	Time (hrs)	Differentiation	Ref.
2D Natural	Collagen	hMSCs	5	24	Myogenic	[19]
		hMSCs	10	24	Myogenic^*∗*^	[20]
		hMSCs	3, 10	8, 48	Tenogenic (10%)	[21]
		hMSCs	0.8, 5, 10, 15	48	Osteogenesis (<5%) Osteogenic inhibition (>5%)	[22]

2D Natural + synthetic	Elastin	hMSCs	10	24	Myogenic^*∗*^	[20]
	Silicone	rMSCs	10	N/A	Tenogenic	[23]
	Elastin-silicone	rMSCs	5, 10, 15, 20	24	Myogenic (10%)	[24]
	Elastin-silicone	rMSCs	10	24, 48, 72	Myogenic	[24]

^*∗*^Myogenic expression transiently increased; expression reduced to basal levels after cell alignment.

**Table 3:** Effect of cyclic tensile strain on MSCs within 3D scaffolds.

Scaffold		Cell	Strain (%)	Time (hrs)	Differentiation	Ref.
3D Natural	Collagen	hMSCs	10, 12	28, 56	Osteogenic	[25]
		hMSCs	10	56	Osteogenic	[26]
		hMSCs	10	168	Tenogenic	[27]
		rbMSCs	2.4	96	Inconclusive	[28]

3D Synthetic hydrogel	(RGD/RGE)	hMSCs	3	2, 24	Gene expression ↑ (RGD)	[29]
	PLA	hMSCs	2, 5	15	Inconclusive	[30]
	PEG	hMSCs	10	168	Tenogenic	[31]
	OPC	hMSCs	10	252	Tenogenic	[32]
